# Antioxidant biocompatible composite collagen dressing for diabetic wound healing in rat model

**DOI:** 10.1093/rb/rbab003

**Published:** 2021-03-13

**Authors:** Bei Qian, Jialun Li, Ke Guo, Nengqiang Guo, Aimei Zhong, Jie Yang, Jiecong Wang, Peng Xiao, Jiaming Sun, Lingyun Xiong

**Affiliations:** 1 Department of Plastic Surgery, Union Hospital, Tongji Medical College, Huazhong University of Science and Technology, 1277 Jiefang Avenue, Wuhan 430022, China; 2 Wuhan Clinical Research Center for Superficial Organ Reconstruction, Wuhan 430022, China

**Keywords:** collagen, graphene oxide, N-acetylcysteine, sustained release, diabetic wound healing

## Abstract

Associated with persistent oxidative stress, altered inflammatory responses, poor angiogenesis and epithelization, wound healing in diabetic patients is impaired. N-acetylcysteine (NAC) is reported to resist excess reactive oxygen species (ROS) production, prompt angiogenesis and maturation of the epidermis. Studies have revealed that graphene oxide (GO) can regulate cellular behavior and form cross-links with naturally biodegradable polymers such as collagen (COL) to construct composite scaffolds. Here, we reported a COL-based implantable scaffold containing a mixture of GO capable of the sustained delivery of NAC to evaluate the wound healing in diabetic rats. The morphological, physical characteristics, biocompatibility and NAC release profile of the GO-COL-NAC (GCN) scaffold were evaluated *in vitro*. Wound healing studies were performed on a 20 mm dorsal full-skin defect of streptozotocin (STZ)-induced diabetic rats. The injured skin tissue was removed at the 18th day post-surgery for histological analysis and determination of glutathione peroxidase (GPx), catalase (CAT) and superoxide dismutase (SOD) activity. In diabetic rats, we confirmed that the GCN scaffold presented a beneficial effect in enhancing the wound healing process. Additionally, due to the sustained release of NAC, the scaffold may potentially induce the antioxidant defense system, upregulating the expression levels of the antioxidant enzymes in the wound tissue. The findings revealed that the antioxidant biocompatible composite collagen dressing could not only deliver NAC *in situ* for ROS inhibition but also promote the wound healing process. This scaffold with valuable therapy potential might enrich the approaches for surgeon in diabetic wound treatment in the future.

## Introduction

Diabetes mellitus (DM) is a chronic metabolic disorder, affecting more than 340 million people, with nearly 20% of them developing diabetic wounds globally [1]. Based on a previous report, in every 30 s, one leg is amputated as a result of impaired wound healing in diabetic patients worldwide [2]. Owing to the absence of measures for prevention, non-healing and poorly healing wounds pose a serious global health threat with elevated DM prevalence [3]. The critical cause of the poor healing of the diabetic wound is prolonged inflammation, poor vascularization and epithelization, and deficient collagen deposition [4]. Additionally, the sustained reactive oxygen species (ROS) production would further delay the recovery of wounds in diabetic patients [3, 5]. Thus, the wound dressing biomaterials with the ability to promote diabetic wound healing have been widely studied.

The biomaterials currently used as matrices for wound repair can be classified into natural and synthetic types according to their origin [6]. Due to the characteristics of easy degradation, remarkable biocompatibility, reduced inflammatory responses and non-immunogenic nature, natural biomaterial has been widely recommended, such as collagen (COL) [7]. COL, one of the main extracellular matrix (ECM) protein factors, potentially regulates the cell phenotype and is involved in the modification of physicochemical characteristics of the scaffold [8]. Moreover, benefits from the chemical groups, abundant in the branches of the molecular structure, COL is reported to be the vehicle cross-linked multiple drugs [9]. Unfortunately, poor mechanical properties, the unstable porous structure under wet conditions, and lack of biological activity limit the application of pure COL scaffolds [10].

Graphene oxide (GO), a highly oxidized form of graphene, in which the quasi-two-dimensional (2D) spatial structure of carbon atoms is decorated with oxygen-containing functionalities [11]. Numerous oxygen-functional groups, including carboxylic, epoxy and hydroxyl groups make its surface open for covalent, electrostatic and hydrogen bonding with biomolecules and therapeutic drug, which endows drugs with targeted and sustained features, and improves the biocompatibility of GO [12]. Studies have reported that GO has the potential to promote cellular proliferation, differentiation and adhesion with no or little cytotoxic effect [13, 14].

Moreover, the inclusion of GO nanosheets can substantially improve the mechanical strength to GO-related polymeric scaffolds [15]. After implantation into the body, scaffolds with GO are degraded via peroxidase secreted by immune cells; eventually, their excretion occurs in the form of feces and urine [16]. Thus, owing to its remarkable biocompatibility, biodegradation and mechanical properties, polymeric scaffolds incorporated with GO hold great potential for tissue engineering applications [17].

N-acetylcysteine (NAC) is a transformed version of cysteine (amino acid), which can permeate cell membrane and rapidly hydrolyze to give cysteine, a precursor of glutathione (GSH). By restoring the intracellular natural antioxidant glutathione levels, NAC assists the cells in eliminating the damaging impacts of the ROS along with a high safety profile [18]. As an antioxidant, NAC has been receiving growing research attention in promoting wound healing [19]. Recently, Ozkaya *et al.* [20] reported using a diabetic rat model that NAC improved healing of wounds by reducing the oxidative stress parameters in both tissue and serum. Zayed and colleagues [21] found that NAC accelerated amputation stump healing not only through resisting oxidative stress of the wound but also by promoting vascularization. There are also reports that NAC promotes the expression of matrix metalloproteinase 1 (MMP-1) through the PKC/Stat3 signaling pathway, thereby promoting epidermal maturation [22]. Besides, the carboxyl group of NAC has been confirmed to be chemically cross-linked to the amino group or hydroxyl group, thereby achieving sustained release of NAC [23]. This would ensure adequate drug concentration on the wound, especially in cases of prolonged healing.

Herein, a novel N-acetylcysteine-controlled-release graphene oxide-collagen scaffold (GO-COL-NAC) was developed with 2 mm height and 20 mm diameter, which cross-linked by the carbonized diimine (EDC) and n-hydroxy succinimide (NHS) systems. The surface morphological features of the hybrid scaffolds were assessed using scanning electron microscopy (SEM). To characterize the scaffolds, we used Fourier transform infrared (FTIR), X-ray diffraction (XRD), Raman spectroscopy and spectroscopy. The physical properties, biocompatibility of this scaffold and its ability to release drugs sustained were assessed *in vitro*. Then, the constructed scaffolds were implanted into a rat model having a skin defect (20 mm full-thickness) on the dorsal side, to evaluate the GO-COL-NAC (GCN) scaffold effect on wound healing in the streptozotocin-induced diabetic rats.

## Materials and methods

### Preparing GO-COL and GO-COL-NAC composite scaffolds

**Figure 1.**
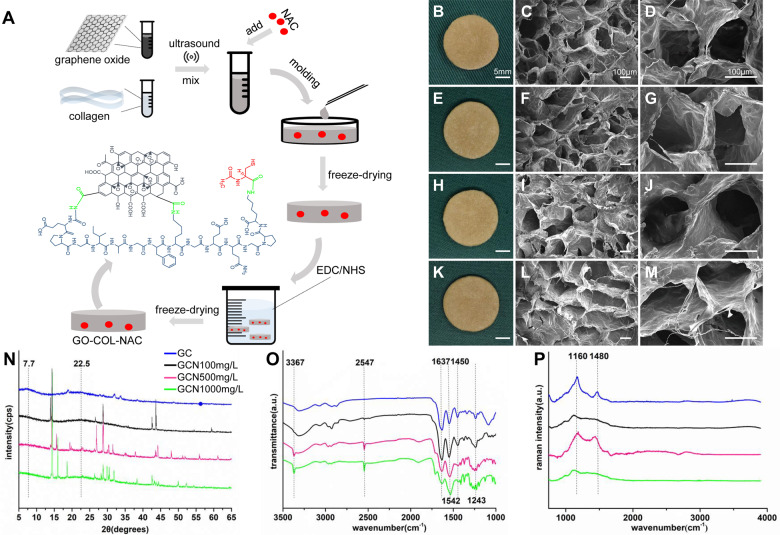
Characterizations of engineered hybrid scaffolds. (**A**) Schematic illustration showing how GO-COL-NAC scaffolds were prepared. GO and COL were dissolved, adequately under an ultrasonic wave. Add the NAC and mix again, the mixture was filled into molds to generate GO-COL-NAC scaffolds with cylindrical shape with 20 mm diameter and 2 mm height. After freeze-dried, cross-linked by EDC/NHS and freeze-dried again, the GO-COL-NAC scaffolds were prepared. Macroscopic view and SEM images of the GO-COL (**B**, **C–D**), GO-COL-NAC100 mg/L (**E**, **F–G**), GO-COL-NAC500 mg/L (**H**, **I–J**) and GO-COL-NAC1000 mg/L (**K**, **L–M**). (**N**) X-ray diffractometer, (**O**) Fourier infrared spectrometer, black dashed line in 2547 cm^−1^ indicates the characteristic peak of NAC and (**P**) Raman spectroscopy, dashed lines in 1160 nm and 1480 nm show the specific peak of GO

Firstly, type I COL (Sigma-Aldrich, America) was cut into pieces, dissolved in acetic acid (0.05 M) to generate a 2% (w/v) suspension. Secondly, upon dissolving GO (Sigma-Aldrich, USA) in distilled water, we sonicated the solution for 30 min using an ultrasonic processor (Branson, USA) to generate a 0.2% (w/v) solution. Thirdly, the GO solution was mixed with COL suspension completely in a 1:5 volume ratio and added NAC (Sigma-Aldrich, USA) (500 mg/L) aqueous solution. The obtained mixture was then sonicated at 70% intensity in an ice bath for 60 min to obtain a homogeneous state. Fourthly, the mixed solution was filled into a pre-designed cylindrical model (20 mm, diameter; 2 mm, height). We then froze the solution at −20°C overnight. This was followed by a 24 h lyophilization at −50°C, after which we obtained a porous scaffold. Finally, we cross-linked the scaffolds chemically through immersion into EDC (50 mM) (Sigma-Aldrich, USA) with NHS (20 mM) (Sigma-Aldrich, USA) solution (H_2_O/ethanol = 5:95) for 24 h, adequately washed using distilled water, then freeze-dried at −50°C; resulting in a cross-linked GO-COL-NAC composite scaffold (Fig. 1A). We fabricated the control GC, GCN100 mg/L and GCN1000 mg/L scaffolds through the same methods, but the added NAC concentration was 0, 100 mg/L and 1000 mg/L, respectively.

### Characterizing GO-COL and GO-COL-NAC composite scaffolds

#### Morphology of GO-COL and GO-COL-NAC composite scaffolds

The SEM (JSM-IT300, JEOL, Tokyo, Japan) run at a high voltage (20 kV) was used to characterize the morphology of the GO-COL and GO-COL-NAC scaffold specimens after sputter-coating them with gold–palladium.

### Water absorption rate, water retention ratio and porosity

The water absorption rate and retention rate of the scaffolds were measured as reported previously [24]. Briefly, dry scaffolds were weighed (*W*_1_), immersed in distilled water at room temperature for 24 h. Using a filter paper, the surface water was absorbed, and the wet weight was designated as *W*_2_. Upon filling the scaffolds with water, the tubes were centrifuged (500 rpm, 3 min) then re-weighed (*W*_3_). To calculate the percentage weight absorption and retention rate of the GO-COL or GO-COL-NAC scaffold, we utilized the following equation.

$$\mathrm{Water}\;\mathrm{absorption}\;\mathrm{rate}\;\left(\%\right)\;=[(W_{2}-\;W_{1})/W_{1}]\;*100\%$$

 

$$\mathrm{Water}\;\mathrm{retention}\;\mathrm{rate}\;\left(\%\right)=[(W_{3}-\;W_{1})/W_{1}]\;*100\%$$

The porosity of the scaffolds was determined according to the modified liquid displacement method reported in the previous study [25]. Firstly, the following measurements were taken: the radius (*r*), thickness (*h*), and dry weight (*W*_d_) of the scaffold. Secondly, the scaffold was immerged into absolute ethanol for 24 h at room temperature, then taken out and immediately weighted as *W*_w_. Each of the above values was given based on the average of three parallel specimen measurements. The following formula was used to calculate the porosity of the GO-COL or GO-COL-NAC scaffold:

$$\mathrm{Porosity}\;\left(\%\right)=\;[(W_{w}-W_{d})/(\rho h\pi r^{2})]*100\%$$

where ρ represents the absolute ethanol density (0.789 g/cm^3^), and the π value is 3.14159.

### X-ray diffraction, Raman spectra analysis and Fourier transform infrared spectroscopy

X-ray diffraction (XRD, Empyrean, PANalytical B.V. Netherlands) with Cu Kα radiation (scanning rate: 1.4583°/s, 2θ range from 5° to 65°) was used to evaluate the crystalline phases of the scaffolds. Raman microscope (Rfs 27 FT, Bruker, Germany) was applied to examine the composition of the scaffolds; the scanning range was 50–4000 cm^−1^ at room temperature. The excitation source is a diode laser with wavelength (532 nm) and power (30 mW) [26]. Functional groups of the scaffolds were confirmed using Fourier transform infrared spectroscopy (FTIR, VERTEX 70, Bruker, German) (4000–500 cm^−1^) with 2 cm^−1^ resolution.

### The release kinetics of NAC in GO-COL-NAC scaffolds

The drug release properties of the scaffolds were using high-performance liquid chromatography (HPLC, Agilent, USA) using a C18 chromatographic column measuring 200 mm × 4.6 mm, 5 μm The mobile phase contained 20 mmol/L sodium dihydrogen phosphate solution (adjusted pH to 2.5 with 50% phosphoric acid)-acetonitrile (v/v, 94:6) run at a flow rate of 1.0 ml/min. The injection volume was 10 μL, and 210 nm was set as the detection wavelength. Firstly, NAC solutions with different standard concentrations were prepared with a phosphate-buffered solution (PBS). The characteristic peak of NAC was detected by HPLC, and the peak height of the characteristic peaks at different concentrations was recorded. Then a simple unary linear regression between the standard concentration and peak height was established. Secondly, the GO-COL-NAC scaffold was immersed in 2 ml PBS at 37°C with shaking at 60 rpm. The supernatants were collected from the scaffold following centrifugation at 3000 rpm for 5 min at each scheduled time-points (6 h, 12 h, 1 day, 3 days, 7 days, 14 days, 18 days), respectively, and subsequently detected by HPLC. The released NAC was calculated according to the acquired unary function. The experiments were performed in triplicate.

### *In vitro* cell biocompatibility of the GO-COL and GO-COL-NAC scaffolds

NIH 3T3 fibroblasts (ATCC, USA) were used for cell proliferation assay and HaCaT cells (ATCC, USA) for cell migration assay. The cells were maintained in growth media (GM) enriched with Dulbecco’s modified Eagle’s medium (DMEM, Thermo Fisher Scientific, USA) High glucose containing fetal bovine serum, 10% (FBS, Thermo Fisher Scientific, USA), and penicillin/streptomycin, 1% (Thermo Fisher Scientific, USA) at 37°C and 5% CO_2_ in a humidified incubator. For cell detachment, we used 0.05% trypsin, but replaced the media every 2 days.

The scaffolds sterilized using ethylene oxide gas were immersed in the DMEM high glucose medium for 24 h. Subsequently, we collected the media, centrifuged for 5 min at1000 rpm, then filtered them through a 0.20 μm syringe filter. The filtered media as the extract liquor of the scaffold were collected for cytology experiments [27].

### *In vitro* cell proliferation assay

The cell proliferation assay was used to estimate the cytotoxicity of the scaffold according to the protocol previously described [28]. The NIH-3T3 fibroblasts were cultured up to confluency and seeded in a 24-well plate 2 × 10^4^ cells/well followed by incubation for 24 h. Subsequently, the medium in the 24-well plate was respectively replaced with four different extract liquors, low glucose medium and high glucose medium in six groups. The plate was subjected to the IncuCyte S3 Live-Cell Analysis System (Essen Bioscience) where real-time images were captured every 8 h. The proliferation phase was assessed using IncuCyte S3 Software (Essen Bioscience) and compared between groups.

### Real-time polymerase chain reaction analysis for collagen

The mRNA expression levels of COL Type I and COL Type III of the NIH-3T3 fibroblasts in different extract liquor were measured using real-time polymerase chain reaction (RT-PCR). Total RNA extraction from the cultured NIH-3T3 fibroblasts was performed using Trizol reagent (Thermos Fisher Scientific, USA) and reverse-transcribed using the Revert Aid First Strand cDNA Synthesis Kit (Thermos Fisher Scientific, USA). The primer sequences used are shown in supporting information, as in Supplementary Table S1. For RT-PCR, we used using AceQ Universal SYBR qPCR Master Mix (Vazyme Biotech Co., Ltd) on a real-time PCR system (Stepone plus, ABI, USA) for PCR product quantification. Amplification conditions were as follows: 95°C for 5 min, 95°C for 10 s, and 60°C for 30 s (40 cycles). mRNA expression level in all experimental groups was determined using the 2^−ΔΔCT^ method.

### *In vitro* cell migration (scratch) assay

The keratinocyte migration assay was conducted for *in vitro* evaluation of re-epithelialization. HaCaT cells were seeded in 24-well plates with a density of 2.5 × 10^5^ cells in each well and cultured to form a confluent monolayer. Next, add the mitomycin with a concentration of 10 μg/ml and incubate for 3 h. Scratch wounds were created on the cell monolayers using a 200 μL pipette tip and rinsed with PBS gently. Thereafter, with a different extract liquor, we substituted the medium in each well. At 4 h, 8 h, 12 h, 16 h and 24 h of incubation, the cells were subjected to PBS wash, after which images of the scratched wounds were taken. Measurement of the area of the remaining scratched wound at each time-point was taken using Image J software. The setting of the control group is the same as the cell proliferation assay.

### *In vivo* wound healing studies

Study protocols were approved by the Animal Ethical Committee of Huazhong University of Science and Technology (HUST; all animals were obtained from the Laboratory Animal Center of HUST).

Sixteen male 6-weeks-old Sprague−Dawley (SD) rats, with an average weight of 180–200 g were maintained adaptively in a room with controlled humidity and temperature for one week. After fasting overnight, we injected all rats with streptozotocin (STZ, Sigma-Aldrich, USA, 50 mg per kg body weight) in the right upper abdomen. The SD rats with the fasting glucose level > 11.1 mmol/L for three consecutive days were considered DM. Otherwise, STZ was injected again until the DM model was successfully constructed.

To anesthetize the 8 diabetic rats, 10% chloral hydrate (Sigma-Aldrich, USA, 0.4 ml per 100 g) was used via intraperitoneal injection, and their dorsal hairs were shaved. The incision was marked with methylene blue and subsequently disinfected with iodophor. Five full-thickness wounds (20 mm diameter) was randomly created at either side of the dorsal central line in experimental animals. Wounds in each rat were treated with the following: the blank control, GC, GCN100 mg/L, GCN500 mg/L and GCN1000 mg/L randomly; then the wound area was fixed with a medical bandage. The bandage was changed every two days. Using a Canon digital camera, wound images were recorded at days 0, 3, 7, 14 and 18 post-surgery. Image J software was used to quantify the surface area of the wound at each time-point. Then, to calculate the rate of wound closure, we used the following formula:

$$\mathrm{Wound}\;\mathrm{closure}\;\mathrm{rate}=(S_{0}-S_{x})/S_{0}*100\%$$

Where *S*_0_ refers to the original wound area and *S*_x_ denotes the area of the wound at days 0, 3, 7, 14 and 18 post-surgery.

### Histological analysis

All the experimental rats were sacrificed at day 18 post-surgery, and tissues including both unwounded and wounded areas were collected and then fixed in 10% formaldehyde. Hematoxylin–Eosin (H&E) and Masson trichrome stains (Sigma-Aldrich, USA) of wound tissue 18 days post-surgery were performed separately to evaluate the epidermis, collagen and new tissue generation. Similarly, the immunohistochemical stain with antibodies against human-specific CD31 (Sigma-Aldrich, USA) was implemented to observe the vascular endothelial cells [29]. An optical microscope (Nikon H600L, Tokyo, Japan) was used to examine the cells and take images.

### Detection of antioxidant stress level

The relative mRNA expression of various antioxidant kinases, including catalase (CAT), superoxide dismutase (SOD) and glutathione peroxidase (GPx) in skin wound skin samples was determined by RT-PCR analysis according to the protocol described above. The following amplification conditions were used: 95°C for 5 min, 95°C for 10 s and 60°C for 30 s (40 cycles). A list of primers used for PCR is provided in Supplementary Table S2.

## Statistical analysis

We presented all data from the repeated experiments as the mean ± standard deviation, then assessed using Tukey’s *t*-tests and one-way ANOVA (SPSS software, USA; GraphPad Prism, USA). The significance levels were denoted as follows: *, **, and ***, referring to the *P* values of <0.05, <0.01 and <0.001, respectively.

## Results

### Preparation and characterization of GO-COL and GO-COL-NAC scaffolds

The cylindrical GO-COL and GO-COL-NAC scaffolds with a diameter of 20 mm and a height of 2 mm were successfully prepared after crosslinking and re-lyophilizing procedures. As depicted in Fig. 1B, E, H, K, various scaffolds prepared showed the same appearance of a brown sponge. SEM micrographs displayed that the morphologies of GO-COL and GO-COL-NAC scaffolds were both highly interconnected porous under different magnifications (Fig. 1C–D, F–G, I–J, L–M).

The quantitative results of the water absorption rate, water retention rate and porosity about the scaffolds were presented in Supplementary Table S3, which exerted a crucial role in the cell ingrowth and metabolite [30]. No significant difference was found between the three parameters of each group, indicating that the similarity in physiochemical properties, and the concentration of NAC has no effect on the structure of the scaffold.

Figure 1N shows the XRD analysis of each sample. When 2θ = 22.5° and 7.7°, the broad diffuse peak was presented, which could be attributed to the homogeneous COL and GO, respectively. However, the characteristic narrow diffraction peak seen on the NAC-loaded samples may represent the NAC in the form of a microcrystal phase.

The Functional groups of the scaffolds were confirmed by FTIR (Fig. 1O). The appearances of amide I (C = O stretch) around 1637 cm^−1^, amide II (N–H stretch) around 1542 cm^−1^, and amide III (C–N stretch) around 1450 cm^−1^, were dedicated to the COL bands in all four groups [9]. Meanwhile, the characteristic peaks of the GO at 3367 cm^−1^ were observed [31]. The C–O–C stretching corresponded to the peak around 1233 cm^−1^, and the sulfhydryl group (–SH) stretching (at 2547 cm^−1^) was specifically seen in all NAC-loaded samples [26].

Using the Raman spectrum (Fig. 1P), the carbon components were revealed in the four scaffold groups. Notably, four sample groups presented the characteristic ‘M’ peak composed of D band (around 1160 cm^−1^) and G band (around 1480 cm^−1^) (Fig. 1P), which was emitted by the vibration of sp2 hybridized carbon atom plane, indicating the existence of GO in the composite scaffolds.

### The release kinetics of NAC in GO-COL-NAC scaffolds

Here, we have presented the release profiles for the GO-COL-NAC scaffolds in Fig. 5C, demonstrating that the NAC loaded in the scaffolds could be persistently released for at least 18 days. The curves of the three groups were basically identical, containing two stages. The NAC was released rapidly in the first 24 h, about 51.96 ± 1.35% (51.9 ± 1.35 μg) of the total loaded drug in the GCN100 mg/L, 49.42 ± 0.35% (247.1 ± 1.75 μg) in GCN500 mg/L and 49.74 ± 1.63% (497.4 ± 16.3 μg) in GCN1000 mg/L groups. Between 24 h and 14 days, the release rate slowed down, obviously. The cumulative released drug percentage of the GCN100 mg/L was about 72.89 ± 0.75%, the GCN500 mg/L was72.77 ± 0.50%; the GCN1000 mg/L was 71.05 ± 0.86%. The average released dosage of the three groups in the last 4 days was 3.23 ± 0.52 μg, 12.85 ± 1.07 μg and 29.24 ± 3.58 μg, respectively.

### *In vitro* cell biocompatibility of the GO-COL and GO-COL-NAC scaffolds

**Figure 2.**
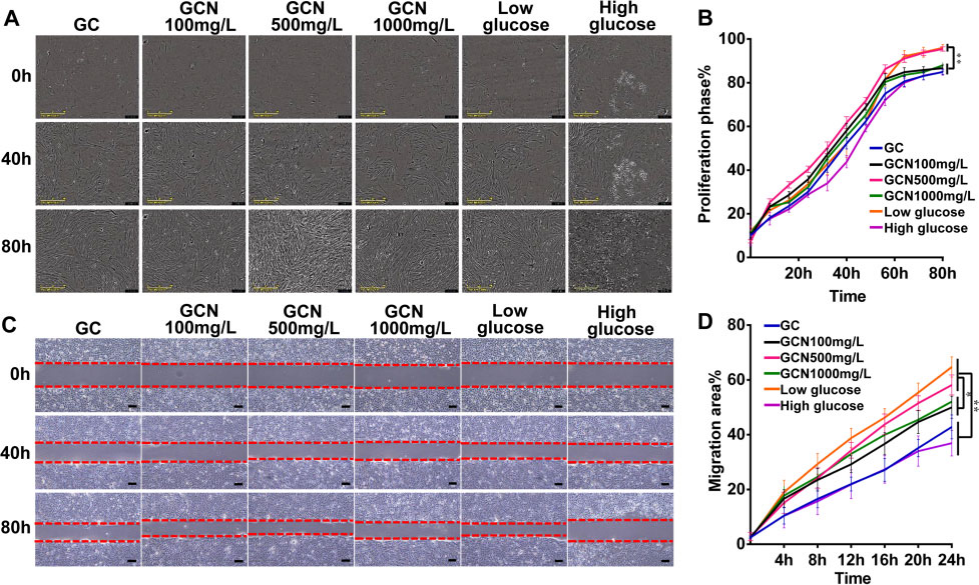
In vitro cell biocompatibility of GO-COL-NAC hybrid scaffolds. (**A**) Images of cell proliferation assay of the NIH 3T3 fibroblasts cultured by extract liquor of different scaffolds at different time-points, observed by the IncuCyte S3 Live-Cell analysis system (scale bars: 200 μm). (**B**)Cell proliferation phase (%) of the NIH 3T3 fibroblasts. (**C**) Scratch assay experiment images of HaCaT cells cultured by extract liquor of different scaffolds at different time-points, observed under a light microscope (scale bars: 100 μm). (**D**) Migration area (%) of the HaCaT cells at different time-points. (*P < 0.05, **P < 0.01)

Cell proliferation of the NIH-3T3 fibroblasts was shown in Fig. 2A. It could be observed that there were more cells in all groups upon increasing culture time for 80 h post-seeding, which suggested the scaffolds had no obvious cytotoxicity. While the GCN500 mg/L group and the negative control group (low glucose) exhibited higher living cell density at 80 h, confirming the better cell proliferation viability compared to the other groups. Some cells in the positive control group (high glucose) had lost their bioactivity, and cells in other groups remain active, although the density of the living cells was relatively low. The NIH-3T3 fibroblasts proliferation phase (Fig. 2B) of the GCN500 mg/L group and the negative control group reached 95.44 ± 1.0% and 96%±1.45% respectively, remarkably higher compared to the other groups; notably, the difference exhibited statistical significance (*P* < 0.01).

The RT-PCR was performed to detect the COL gene expression of NIH-3T3 fibroblasts in the extract liquor of scaffolds. Results (Fig. 6A and B) indicated that the GCN500 mg/L group showed a significantly higher gene expression of the COL Type I (1.81 ± 0.05 fold) and COL Type III (1.95 ± 0.06 fold) compared with other groups.

These outcomes demonstrated that the scaffolds had good biocompatibility and had a better ability to improve COL secretion potential of fibroblasts in a high-glucose environment when NAC concentration was 500 mg/L (*P* < 0.05).

The result of the keratinocyte migration (scratch) assay *in vitro* was shown in Fig. 2C and D. Since keratinocytes were the main cell types that contributed to the wound healing, this general cell migration trend (Fig. 2C) partly reflected the potential of the scaffolds to re-epithelialize *in vitro* [27]. The quantitative analysis (Fig. 2D) using the Image J and Graphpad Software revealed that the HaCaT cell migration rate of each group at 24 h were 42.83 ± 4.36% (GC group), 50.01 ± 3.99% (GCN100 mg/L group), 58.17 ± 3.67%(GCN500 mg/L group), 52.08 ± 2.23%(GCN1000 mg/L group), 64.73 ± 3.66% (low glucose group, negative control), 36.91 ± 4.68% (high glucose group, positive control), respectively. Obviously, the GCN500 mg/L group had a faster cell migration rate in the experimental group (*P* < 0.05), only slightly slower than that of the negative control group (*P* > 0.05).

### *In vivo* wound healing studies

**Figure 3.**
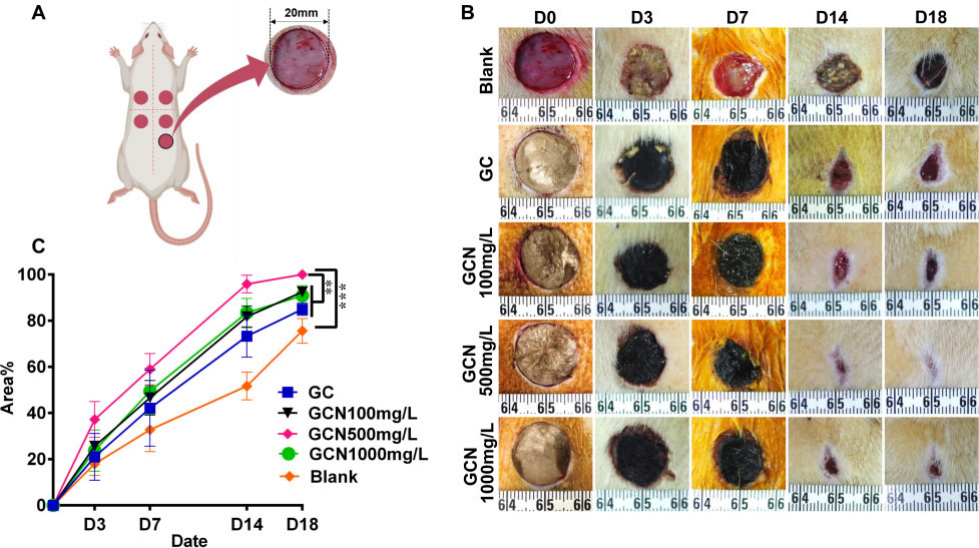
The *in vivo* implantation experiments showing that the GO-COL-NAC500 mg/L scaffold possessed the optimal treatment effects across the five groups. (**A**) Schematic illustration of five 20 mm diameter, full-thickness wounds created at either side of the dorsal Central line. (**B**) Representative photographs of cutaneous wounds at day 0, 3, 7, 14, 18 post-surgery. GO-COL-NAC500 mg/L group has completely healed at day 18 post-surgery, while other groups have not. (**C**) Wound closure rates were shown at days 3, 7, 14, 18 post-surgery. (mean ± SD; ***P < 0.001, **P < 0.01, *n* = 8 per group)

There was a certain mortality rate in the process of diabetic modeling (2 deaths per 10 rats), and an increase in blood sugar, and a decrease in STZ-injected rat’s weight. Finally, eight rats with fasting blood glucose greater than 11.1 mmol/L for three consecutive days were included *in vivo* experiment. Five full-thickness wounds (20 mm diameter) were created at in each diabetic rat to compare the wound healing efficiency between four scaffolds and one blank control, and Fig. 3A was the schematic of the experiment. Fig. 3B shows the macroscopic images of the dorsal wounds at days 0, 3, 7, 14 and 18 post-surgery. In the first 7 days, all the scaffolds adhered well on the wound surface, and there seemed to be no significant difference in the healing rates of all wounds. Subsequently, the scaffolds were degraded subcutaneously, and the wound area of each group showed a large difference. The quantitative analysis shown in Fig. 3C further reveals this trend. On the 18th day, all the wounds treated by the GCN500 mg/L scaffolds presented an almost complete wound closure (100%), remarkably faster than that of group GC (84.97 ± 1.01%), group GCN100 mg/L (92.54 ± 0.77%) and group GCN1000 mg/L (90.86 ± 1.77%), while the healing rate of the blank control was only 75.63 ± 2.18% at the lowest (*P* < 0.05).

**Figure 4.**
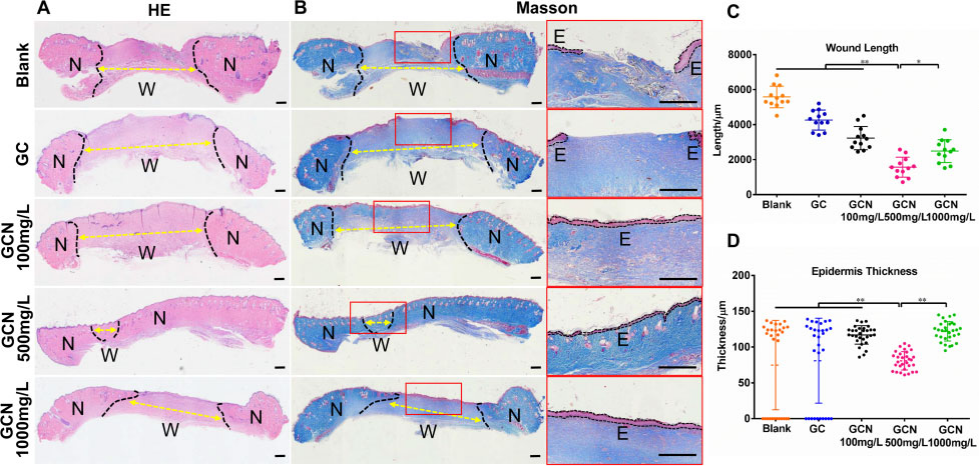
Histological analysis revealing better exhibited better repair effect in the GO-COL-NAC500 mg/L group at day 18 post-surgery compared with other groups. (**A**) H&E staining and (**B**) Masson staining images of the wound section were presented at day 18 post-surgery. The black dotted line represents the interface between the incomplete wound area and the neo-epidermis, the yellow arrows indicate the remaining wound length. W, remaining wound area; N, neo-epidermis; E, epidermis. Scale bar = 500 μm. (**C**) Length of the remaining wound and (**D**) thickness of regenerated epidermal were analyzed (**P < 0.01, *P < 0.05)

HE staining of wound tissue 18 days after surgery was shown in Fig. 4A. The scaffolds implanted were fully absorbed; however, its outline was indistinguishable. The newly generated skin tissue was found in the defect areas. However, the blank control group still presented remarkable tissue defects, where microstructure was disordered and inflammatory cell was infiltrated. The wound treated by GCN500 mg/L scaffolds had been repaired almost completely with the continuous epidermis, whereas the other groups resulted in defect healing with discontinuous epidermis. Masson’s Trichrome staining (Fig. 4B) evaluating the collagen deposition demonstrated that the GCN500 mg/L scaffold could better repair the diabetic wound, which exhibited a uniform and thick collagen bundle deposition compared to other groups. Figure 4C was plotted for comparison of the remaining wound length in different groups. The unhealed wound length for GCN500 mg/L (1568 ± 164.2 μm) was shorter than that of GC (4258 ± 165.9 μm), GCN100 mg/L (3229 ± 191.4 μm), GCN1000 mg/L (2487 ± 188.2 μm) and blank control (5584 ± 179.8 μm). Figure 4D was plotted to assess the thickness of the epidermis. The GCN500 mg/L treated group appeared the best epidermal maturation with uniform thickness (80.81 ± 2.36 μm), while blank control and GC group appeared with incomplete epithelization. The thickness of the GCN100 mg/L and GCN1000 mg/L were 116.9 ± 2.36 μm and 121.9 ± 2.48 μm, respectively, which was much thicker than the GCN500 mg/L.

**Figure 5.**
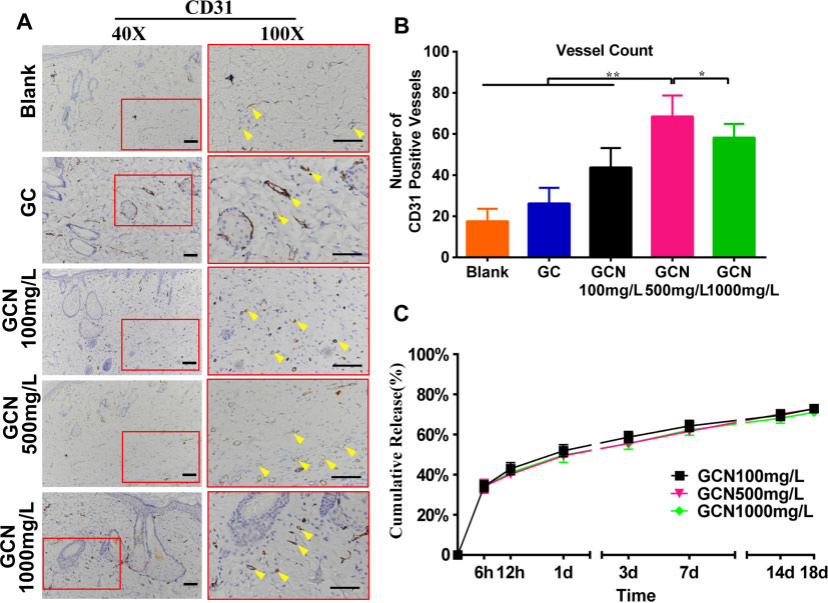
(**A**) Immunohistochemical staining for CD31 of the regenerated skin on day 18 post-surgery showing a significant increase in the number of vessels in the GO-COL-NAC500 mg/L group compared to others. Yellow arrows indicate CD31 positive vascular endothelial cells, Scale bar = 200 μm. (**B**) Quantitative analysis of the number of CD31 positive vessels on the regenerated skin. (***P < 0.001, **P < 0.01). (**C**) The drug release curve of the NAC loaded in GO-COL-NAC scaffolds (*n* = 3 per sample per time-point). The cumulative NAC release increased rapidly in the first 24 days, but still maintained an upward trend until the 18th day, indicating that the GO-COL-NAC scaffold was capable of sustained drug release

CD31 staining (Fig. 5A) was applied to clarify the angiogenesis of the regenerated tissue. The results revealed that the number of blood capillaries in GCN500 mg/L treated group (75.42 ± 3.90) was higher than that of GCN100 mg/L (43.75 ± 2.71), GCN1000 mg/L (55.58 ± 2.35) and blank control (26.25 ± 2.18), which was attributed to the pro-angiogenesis effect of NAC (Fig. 5B).

### Detection of antioxidant stress level

**Figure 6.**
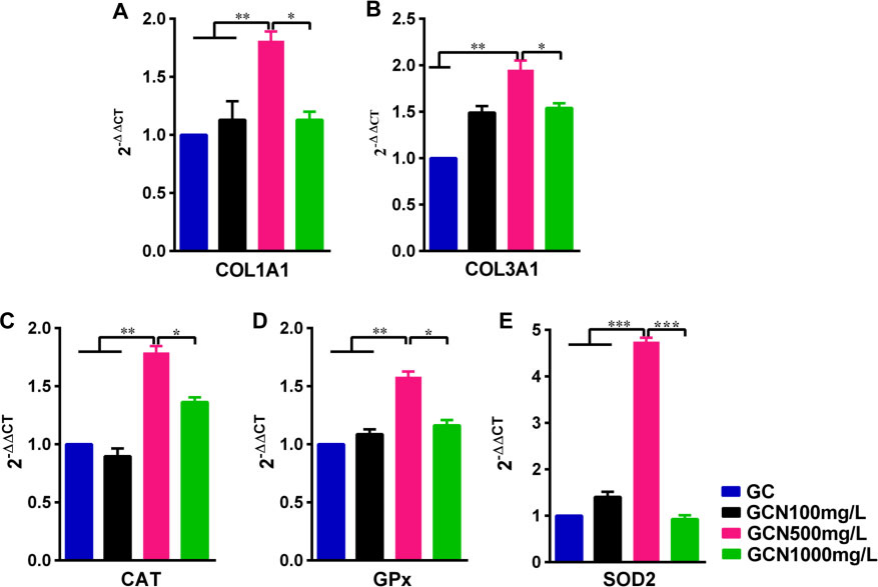
Relative mRNA expressions of COL I (**A**) and COL III (**B**) of the NIH-3T3 fibroblasts in different extract liquor of the scaffolds. The extract liquor from the GO-COL-NAC500 mg/L scaffold induced significantly higher mRNA expression of the COL I and COL III. (**C, D, E**) Analysis of antioxidant kinases gene expression (CAT, GPx and SOD2) of the wound skin sample on day 18 post-surgery revealed that the GO-COL-NAC500 mg/L scaffold exhibited stronger anti-oxidative stress ability than the other two groups (*P < 0.05, **P < 0.01, ***P < 0.001)

The RT-PCR was conducted to monitor mRNA expression of various antioxidant kinases of the wound skin sample on day 18 post-surgery. The results demonstrated that GCN500 mg/L significantly increased the mRNA levels of CAT, SOD and GPx, (Fig. 6C–E), which meant a stronger anti-oxidative stress ability compared to other scaffolds. This effect may benefit from a more appropriate concentration of NAC loading on the scaffold.

## Discussion

A diabetic wound is a common and tiring complication, leading cause of disability and mortality among diabetics [32]. Biomaterials offer promising potential in the induction of tissue regeneration for chronic wound healing. In this study, we fabricated a conducive GO-functionalized COL hybrid scaffold loaded with NAC for wound healing in diabetic rats. It was demonstrated that the GO-COL scaffolds loading 500 mg/L NAC distinctly induced collagen deposition, re-epithelialization and angiogenesis in the wound sites of diabetic rat model; also, it inhibited inflammation reaction. This non-toxic and biodegradable scaffold took advantage of the inherent advantages of GO and COL in promoting tissue regeneration, which could be synthesized by a simple and feasible procedure. It provided locally and sustainably released NAC in diabetic wounds, avoiding both the side effects of systemic drug use and the repetitiveness of topical application.

Diabetic wounds are characterized by severe oxidative stress and persistent inflammatory response, an important cause for which is the excess production of the ROS [33]. Previous studies revealed the activation of nucleotide-binding and oligomerization domain-like receptor family pyrin domain-containing 3 (NLRP3) inflammasome in DM patients via a ROS-mediated pathway [34], which would impair wound healing in mice and humans with type 2 diabetes [35]. Pierce *et al.* [36] concluded that excessive ROS causes damages to cellular membranes, proteins, lipids; the DNA is thus deleterious to wound healing. Additionally, ROS was reported to regulate the synthesis and release of various proinflammatory cytokines, including TNF-α, IL-1β by activating transcription factors, including nuclear factor erythroid 2-related factor 2 (Nrf-2) and nuclear factor kappa B (NF-κB) [37]. In other words, the accumulation of ROS not only participates in the development of diabetic wound but also propagate the inflammatory cascade, which impairs wound healing and hinders the formation of new tissues. Hence, Boniakowski *et al.* [38] declared that therapies that permit regulated and programmed wound inflammation are promising approaches for diabetic management as well as other pathologic wounds.

NAC, a sulfhydryl antioxidant, has been receiving growing research attention in the chronic wound because of its ability to regulate excessive inflammatory response and prevents oxidative stress triggered by ROS [39]. The proven mechanisms of the NAC promoting wound healing are as follows: (i) As a precursor of GSH, NAC increases the synthesis of this antioxidant, which reduces free radical damage. (ii) NAC upregulates the PI3K expression, which is responsible for growth factor and cytokine production necessary for wound healing. (iii) NAC activates the stat3 expression, a protein essential for migration of dermal microvascular endothelial cells to promote angiogenesis. (iv) NAC increases the expression of MMP-1, a protein responsible for ECM formation and re-epithelialization [22, 40]. In the current study, compared with the GC scaffold, GCN500 mg/L scaffold significantly increased the mRNA expression of antioxidant enzymes (CAT, SOD, GPx) in the wound tissues 18 days after surgery (Fig. 6C and D), which was attributed to the NAC. It meant that GCN500 mg/L scaffold exhibited higher levels of antioxidant stress. Meanwhile, due to the angiogenic effect of the NAC, and the amelioration of the wound micro-environment after free radical scavenging, immunohistochemical examination of the wound tissue of GCN500 mg/L scaffold found that there was more angiogenesis than that of the GC scaffold (Fig. 5A and B). Also, the comparison of the GCN500 mg/L and GC scaffold results *in vitro* cell proliferation, cell migration, and cellular COL mRNA expression was also consistent with the above (Figs. 2 and 6A, B). However, the premise of achieving these results is that the NAC-loaded scaffold can release an effective drug concentration on the wound surface sustainably. Therefore, the release kinetics of the NAC loaded in the GO-COL-NAC scaffolds will be a crucial parameter.

As shown in Fig. 5C, NAC-loaded scaffolds present a rapid drug release in the first 24 h, mainly due to free NAC release deposited within the pores of the scaffolds. And then, until the 18th day, the curve shows a slow upward trend, which may be associated with the breakage of chemical bonds between NAC and the scaffold, causing more NAC to be released slowly.

However, the differences in results between the NAC-loaded scaffolds suggest that NAC concentration plays a crucial role. Tsai *et al*. [22] reported a dose-dependent manner with NAC concentrations of 0.1 mM to 1.0 mM by mice burn model. Furthermore, some previous studies have pointed out the specific recommended doses for NAC in dermatologic conditions [41, 42]. In this present research, compared with the NAC loading concentration of 100 mg/L, the GCN500 mg/L treated wound presented a more uniform and thicker collagen bundle deposition, better epidermal maturation with uniform thickness and more angiogenesis (Figs. 4 and 5A). This may be due to the longer maintenance time of effective drug concentration in the wound caused by the higher NAC concentration in a certain range. Nevertheless, 1000 mg/L NAC loading concentration showed relatively poor results in the experiment compared with 500 mg/L, which implies the loading range due to excessive drug concentration. Tsai *et al.* [22] confirmed that the expression of MMP-1 was positively regulated by NAC drugs, and the production of excessive MMP-1 would lead to excessive epidermal hyperplasia, which explained the thickest epidermis in the highest NAC loading group(1000 mg/L) (Fig. 4B). The optimal dose of NAC in topical administration of diabetic wound healing still remains inconclusive; the related research needs to be further carried out.

Additionally, an ideal scaffold using for wound healing should also be endowed with high water absorption and retention capacity, and porosity. Both COL and GO have been shown to have rich hydrophilic groups [43, 44], and the engineered scaffolds demonstrated a distinct porous structure, which ensures the good physical properties of the scaffolds (Supplementary Fig. S1). When applied on the wound, on the one hand, the hybrid scaffold can absorb excess exudate from the wound, and maintain a humid micro-environment locally on the wound, thus conducive to the formation of the granulation tissue. On the other hand, its porous structure facilitates the exchange of oxygen and matter between the cells that grow into the scaffold [30]. Moreover, the previously suspected biotoxicity of GO has not been observed in our study, which may be due to the low concentration of GO in the scaffolds [45], and the modification of COL and NAC to improve its biocompatibility. According to the literature, the toxicity of GO is mainly related to its size, concentration and preparation process, the small-sized and low-concentration GO is usually considered to have good biocompatibility [46].

## Conclusion

In this study, we developed a NAC-loaded GO-COL hybrid scaffold with good biocompatibility and degradability. When the NAC concentration loading on the scaffold is assigned to 500 mg/L, the hybrid scaffold presents a better efficiency to promote diabetic wound healing by resisting oxidative stress, promoting angiogenesis, accelerating ECM-synthesis and facilitating epithelization. This scaffold with valuable therapy potential might enrich the approaches for surgeons in diabetic wound treatment in the future.

## Supplementary Material

rbab003_Supplementary_Data
