# Supplementary material for: Antioxidant biocompatible composite collagen dressing for diabetic wound healing in rat model
Source: Regen Biomater. 2021 Mar 13;8(2):rbab003. doi: 10.1093/rb/rbab003 (PMC7955720; doi:10.1093/rb/rbab003)
Supplement: rbab003_Supplementary_Data [file rbab003_supplementary_data.docx]

**Supplementary material**

**Anti-oxidant biocompatible composite collagen dressing for diabetic wound healing in rat model**

Bei Qian^1,2, a^, Jialun Li^1,2, a^ , Ke Guo^1,2^, Nengqiang Guo^1,2^, Aimei Zhong^1,2^, Jie Yang^1,2^, Jiecong Wang^1,2^, Peng Xiao^1,2^, Jiaming Sun^1,2,^*, Lingyun Xiong^1,2,^*

1. Department of Plastic Surgery, Union Hospital, Tongji Medical College, Huazhong University of Science and Technology, 1277 Jiefang Avenue, Wuhan, 430022, China.

2. Wuhan Clinical Research Center for Superficial Organ Reconstruction, Wuhan 430022, China

a. These authors contributed equally to this work.

* Correspondences authors：

Corresponding authors’ E-mail addresses:

Jiaming Sun: E-mail: 2004xh0801@hust.edu.cn Tel: 027-85726114.

Lingyun Xiong: E-mail: xionglingyun1986@163.com Tel: 027-85726114.

**Figure S1**


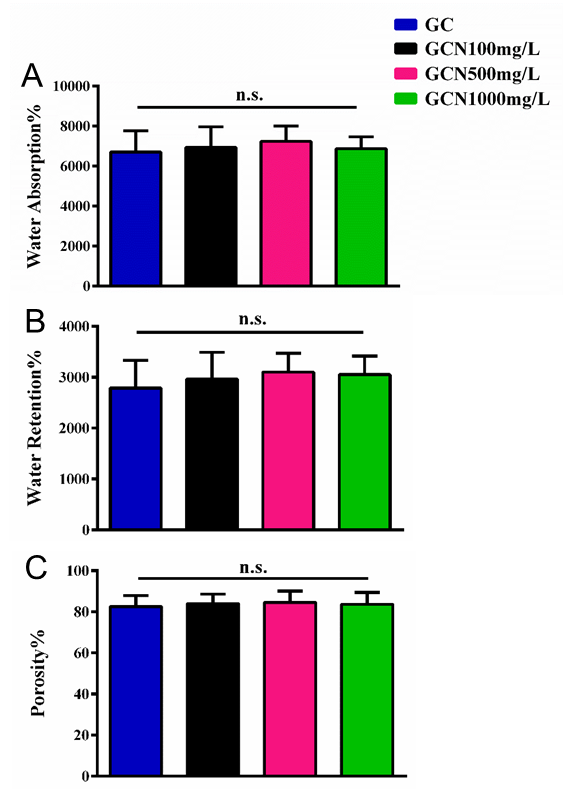


Fig.S1: GO-COL and GO-COL-NAC scaffolds displayed the excellent water absorption, water retention properties and high porosity. The quantitative analysis of the Water absorption (A), water retention (B), porosity (C), (mean ± SD; n=6, n.s. means no significant difference)

**Table S1. Primers used for the real-time PCR analysis；(COL Ⅰ, type I collagen; COL Ⅲ, type Ⅲ collagen)**

| Gene name | Primers name | Primer sequence | Product size (base pairs) |
| --- | --- | --- | --- |
| COL I | H-COL1A1-F  H-COL1A1-R | GATTGACCCCAACCAAGGC;  GAATCCATCGGTCATGCTCT | 178 |
| COL Ⅲ | H-COL3A1-F  H-COL3A1-R | AACGAGAAAAGCGTCAAGCC;  CCAGTCATTCCACCCCACA | 102 |
| β-Actin | R-Actin-F | GTCCACCGCAAATGCTTCTA | 190 |
|  | R-Actin-R | TGCTGTCACCTTCACCGTTC |  |

**Table S2. Primers used for the real-time PCR analysis; (CAT,** **catalase; SOD2, superoxide dismutase 2; GPx1,** **glutathione peroxidase)**

| Gene name | Primers name | Primer sequence | Product size (base pairs) |
| --- | --- | --- | --- |
| CAT | R-Cat-F  R-Cat-R | CCGCCTGGGACCAAACTATC;  GGGGTAGTAGTTGGGAGCAC | 127 |
| SOD2 | R-Sod2-F  R-Sod2-R | ACCGAGGAGAAGTACCACGA;  TGGGTTCTCCACCACCCTTA | 148 |
| GPx1 | R-Gpx1-F  R-Gpx1-R | TTTCCCGTGCAATCAGTTCG;  TCCGCAGGAAGGTAAAGAGC | 167 |
| β-Actin | R-Actin-F | CGTTGACATCCGTAAAGACCTC； | 110 |
|  | R-Actin-R | TAGGAGCCAGGGCAGTAATCT |  |

**Table S3.** **Summary of the physiochemical properties of the GO-COL and GO-COL-NAC scaffolds**

| Scaffold | GC | GCN100mg/L | GCN500mg/L | GCN1000mg/L |
| --- | --- | --- | --- | --- |
| Water Absorption rate(%) | 6703±1064 | 6937±1028 | 7241±766.1 | 6871±591.6 |
| Water retention rate(%) | 2785±548.3 | 2966 ±522.0 | 3103±369.2 | 3050±366.5 |
| Porosity (%) | 82.51±5.393 | 83.99±4.644 | 84.55±5.473 | 83.59±5.875 |
